# New Technique for Probing the Protecting Character of the Solid Electrolyte Interphase as a Critical but Elusive Property for Pursuing Long Cycle Life Lithium-Ion Batteries

**DOI:** 10.1021/acsami.2c11992

**Published:** 2022-09-16

**Authors:** Enrique Garcia-Quismondo, Sandra Alvarez-Conde, Guzmán Garcia, Jesús I. Medina-Santos, Jesús Palma, Edgar Ventosa

**Affiliations:** † Electrochemical Processes Unit, IMDEA Energy, Avda. Ramón de la Sagra 3, 28935 Móstoles, Madrid, Spain; ‡ Department of Chemistry, University of Burgos, Plaza Misael Bañuelos s/n, E-09200 Burgos, Spain; § International Research Centre in Critical Raw Materials-ICCRAM, University of Burgos, Plaza Misael Bañuelos s/n, E-09001 Burgos, Spain

**Keywords:** cycle life, solid electrolyte interphase (SEI), protecting character, coulometry method, redox mediator

## Abstract

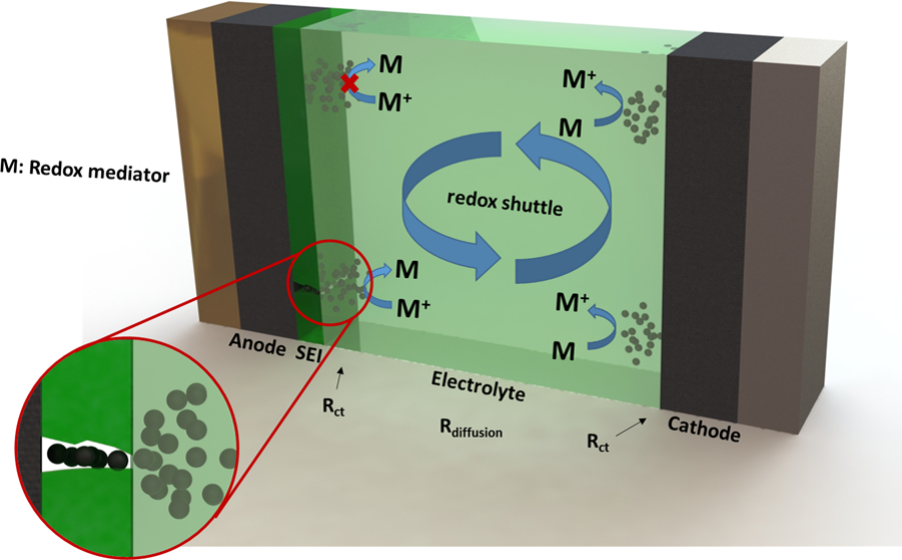

The formation of a protecting nanolayer, so-called solid electrolyte interphase (SEI), on the negative electrode of Li-ion batteries (LIBs) from product precipitation of the cathodic decomposition of the electrolyte is a blessing since the electrically insulating nature of this nanolayer protects the electrode surface, preventing continuous electrolyte decomposition and enabling the large nominal cell voltage of LIBs, e.g., 3.3–3.8 V. Thus, the protection performance of the nanolayer SEI is essential for LIBs to achieve a long cycle life. Unfortunately, the evaluation of this critical property of the SEI is not trivial. Herein, a new, cheap, and easily implementable methodology, the redox-mediated enhanced coulometry, is presented to estimate the protecting quality of the SEI. The key element of the methodology is the addition of a redox mediator in the electrolyte during the degassing step (after the SEI formation cycle). The redox mediator leads to an internal self-discharge process that is inversely proportional to the protecting character of the SEI. Also, the self-discharge process results in an easily measurable decrease in Coulombic efficiency. The influence of vinylene carbonate as an electrolyte additive in the resulting SEI is used as a case study to showcase the potential of the proposed methodology.

## 1 Introduction

Lithium-ion batteries (LIBs) are now extensively used in many applications ranging from powering portable electrics to buffering energy intermittently generated from renewable sources. The growing market of electric vehicles powered by LIBs has triggered a remarkable interest in this battery technology in recent years. The power source of choice for electric vehicles is based on the high energy density, energy efficiency, and moderate cost of LIBs. Nevertheless, the demanding requirements of electric vehicles require further improvements in several aspects of LIBs: safety,^1−3^ cost,^4^ accessibility of raw materials,^5^ recyclability,^6,7^ energy density,^8^ and cycle life.^9,10^

Cycle life is a key performance indicator (KPI) for LIBs, which is especially relevant for electric vehicles due to the impact of replacing the entire battery pack (>10 kWh). As a matter of fact, the cost and cycle life of LIBs are two of the major KPIs that determine the market penetration of electric vehicles.^11,12^ Among the many processes occurring simultaneously in a LIB, electrolyte decomposition is critical for the cycle life since it consumes charges irreversibly, increases the internal resistance, and generates gasses.^13,14^ Since nominal voltages of LIBs are >3.5 V, nonaqueous electrolytes having a wide electrochemical window are needed. The strong reducing conditions required for the lithiation of graphite (state-of-the-art negative electrode material) lead to cathodic decomposition of the electrolyte. Even state-of-the-art carbonate-based electrolytes undergo cathodic decomposition at ca. −2 V vs standard hydrogen electrode (or +1 V vs Li/Li+). Fortunately, the products of the reduction are precipitated on the electrode surface to form a protecting nanolayer, referred to as solid electrolyte interphase (SEI).^15^ This nanolayer SEI is responsible for preventing the continuous decomposition of the electrolyte while allowing Li ions to move through. Thus, ionic and electric properties of the SEI that is formed during the first charging cycles play a critical role in the electrochemical performance of LIBs. Consequently, much effort has been devoted to understanding and improving the SEI.^16,17^

**Figure 1:**
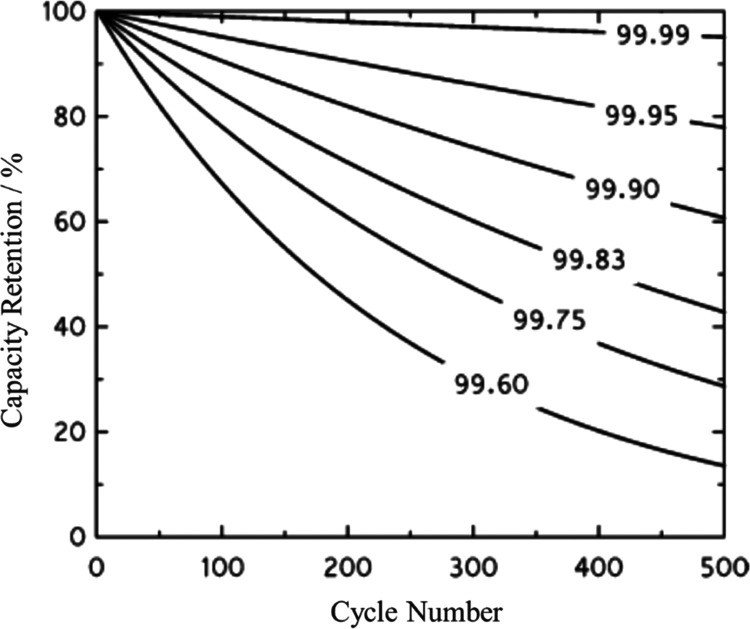
Theoretical evolution of the capacity retention with the number of cycles as a function of the Coulombic efficiency. Adapted with permission.^21^ Copyright © 2014, American Chemical Society.

Ionic properties of the nanolayer SEI are often evaluated by electrochemical impedance spectroscopy (EIS), as the charge-transfer resistance of Li ions across the SEI can be quantitatively determined with this technique. EIS has been widely used to investigate the evolution of the internal resistance as this technique enables deconvoluting the contribution of the SEI as a function of a number of parameters, e.g., number of cycles, electrolyte additive, temperature, etc.^18−20^ However, the evaluation of the electric properties of the nanolayer SEI is not so straightforward. An indirect way of doing this is to analyze the Coulombic efficiency. Since electrolyte decomposition due to a poor-performing SEI results in irreversible consumption of charges, the difference between charges consumed and released during the charge and discharge process, respectively (Coulombic efficiency) can be used to determine how effective is the protecting character of the SEI. Figure 1 shows the influence of the Coulombic efficiency on the retention of energy storage capacity upon cycling, illustrating the critical role of the SEI in the cycle life of LIBs.^21^ Since the cyclability of state-of-the-art LIBs is well above 500 cycles, the cells should operate at Coulombic efficiencies above 99.96%, considering that end-of-life is technically reached when the capacity retention falls below 80%. For high-performance LIBs reaching +2000 cycles, the Coulombic efficiency needs to be >99.99%. Standard electronic equipment, referred to as cycler, cannot reliably provide such values excluding this technique for an accelerated evaluation of protecting the character of the SEI for high-performance LIBs. Thus, the use of standard cycling testing as a tool for improving the cycle life of already high-performing LIBs requires very long periods of time. Dahn’s group, who has largely contributed to this field, proposed a high-precision system to be able to reliably measure values above 99.90% and thus make use of the Coulombic efficiency as an accelerated technique for further improvements in the cycle life of high-performing LIBs. The relevant results and general interest in accelerated methodology led to the creation of a company to commercialize such a high-precision coulometry system. However, this powerful equipment is not accessible to most research battery laboratories.

In this work, a new methodology that is based on coulometry measurements is proposed for accelerated assessment of the protecting character of the nanolayer SEI, which is a critical parameter for the cycle life of high-performance LIBs. The key feature of the methodology is the addition of a redox mediator in the electrolyte after the formation of the SEI (during degassing step). In this way, a charge-transfer reaction between redox mediator and anode across the SEI leads to irreversible loss of charges and thus a decrease in Coulombic efficiency. As a result, the Coulombic efficiency becomes very sensitive to the protecting character of the SEI. Importantly, this methodology enables the use of conventional and low-cost cyclers present in standard battery research laboratories.

## 2 Experimental Section

### 2.1 Preparation of Pouch Cells

Pouch battery cells were assembled using an aluminum case sized 100 × 70 mm, dry electrodes supplied by Custom Cells Itzehoe GmbH, Germany, and microporous separators (Celgard). The pouch cells have a specified capacity of 10 mAh and a voltage range of 1.5–2.1 and 2.5–3.6 V for LTO–LFP cells and graphite–LFP cells, respectively.

Battery cells were filled with different electrolyte formulations using a syringe and vacuum sealed under −90 kPa gauge pressure in a vacuum sealer (TOB-YF200 Vacuum Sealing Machine) for 6 s at 170 °C under a vacuum at −90 kPa of pressure. The cells were allowed to stand for 10–15 min to ensure the full wetting of the electrodes. The cells were then transferred to a 25 °C temperature-controlled box to undergo formation. Pouch cells were charged using a Neware BTS4000 series charger to their upper cutoff voltage at 0.1 C and discharged at 0.3 C. The last charging step at a low current rate of 0.1 C, to complete the formation of the SEI, was carried out. For those batteries assembled with LTO anodes, no previous conditioning protocol has been applied. Therefore, after electrode wetting, the pouches were available for charge and discharge cycles at a nominal current rate (0.3 C).

Then, the batteries were transferred back to an Ar-filled glovebox and degassed (not in the case of LTO anodes). For those pouch cells in which the proposed methodology with redox mediators has been tested, the samples were reintroduced into the glove chamber to add 0.25 mL of the mediator and vacuum sealed again in the same manner as described previously. Finally, the batteries were connected to the battery tester under charging and discharging conditions at a nominal rate of 0.1 C unless otherwise indicated. In the case of not using a mediator, the cyclability of the battery was evaluated under the same nominal conditions (0.1 C in charge and discharge).

The validation of the coulometry methodology started with the evaluation of the redox shuttle functionality of the mediators tested on battery anodes based on the cells assembled with LTO–LFP as electrodes. Then, the coulometry methodology on SEI nanolayers was done experimentally on graphite–LFP pouch cells under different conditions of SEI structuration (adding different proportions of additive), and with different types of mediators. To facilitate discussion, pouch cells prepared without a redox mediator in the electrolyte were denoted as vinylene carbonate (VC)-free and those with s mediator were denoted as VC-free + M (M being the mediator). In a similar way, pouch cells assembled with VC additives in the electrolyte were denoted as “X”VC, where X is the additive concentration in wt %.

The positive electrodes were LiFePO_4_ (1.0 mAh cm^–2^) both from Custom Cells Itzehoe GmbH, Germany. The negative electrodes of these cells were made of artificial graphite (1.1 mAh cm^–2^) and spinel lithium titanium Li_4_Ti_5_O_12_, also from Custom Cells Itzehoe GmbH, Germany.

Redox mediators were selected based on the know-how of our group about nonaqueous redox flow batteries: ferrocene (FC) and methyl phthalimide (PHT) were purchased from Sigma-Aldrich. Due to the more cathodic redox potential of PHT, its redox reversibility is very sensitive to the presence of water. Thus, PHT was vacuum-dried before it was introduced inside the glovebox to eliminate moisture as it was slightly hygroscopic. Table 1 summarizes the main characteristics of the pouch cells used in these investigations.

**Table 1:** Main Characteristics of the Pouch Cells Used in These Investigations

sample	VC [wt %]	mediator	C-rate	code
LTO–LFP	0		0.3 C	VC-free
LTO–LFP	0	FC	0.1 C	VC-free + FC
graphite–LFP	0		0.3 C	VC-free
graphite–LFP	2		0.3 C	2VC
graphite–LFP	0	FC	0.3 C	VC-free + FC
graphite–LFP	2	FC	0.3 C	2VC + FC
graphite–LFP	0	PHT	0.3 C, 1 C	VC-free + PHT
graphite–LFP	2	PHT	0.3 C, 1 C	2VC + PHT
graphite–LFP	6	PHT	0.3 C, 1 C	6VC + PHT

Electrolyte solutions containing VC were prepared by adding the additive in a 1:1 volume ratio of ethylene carbonate (Sigma-Aldrich, Anhydrous, ≥99%): diethyl carbonate (Sigma-Aldrich, anhydrous, ≥99%) with lithium hexafluorophosphate salt (Sigma-Aldrich, battery grade, ≥99 trace metals basis) in an Ar-filled glovebox to ensure minimal exposure to moisture and oxygen. The salt was weighed on an analytical balance and transferred to a vial. The amount of solvent needed to make a 1 M solution was added to the vial using a micropipette, with an accuracy of ±0.01 mL. The vinylene carbonate (VC, Sigma-Aldrich, 99.97%, water content <100 ppm) was added to the electrolyte in amounts of 2 and 6 wt %. Electrolyte without additives is henceforth referred to as VC-free.

### 2.2 Electrochemical Experiments

To evaluate the SEI quality, the Coulombic efficiency of cells was measured using computer-controlled cycling equipment (Neware Electronic Corporation, Shenzhen, China) at 25 ± 0.1 °C inside an ACS Angelantoni climatic chamber (Model DYR250). After performing two SEI formation cycles at 0.1 C, the cells after the formation were tested for six cycles at the specified C-rate between 1.5 and 2.1 V for the LTO–LFP cells and 2.5 and 3.5 V for the graphite–LFP cells.

## 3 Results and Discussion

### 3.1 Concept of Enhanced Redox-Mediated Coulometry for the Evaluation of the Protecting Character of the SEI

The overall protecting character of the SEI determines the extent of cathodic decomposition of the electrolyte upon cycling. The effectiveness of this SEI property can be indirectly estimated via Coulombic efficiency since the extent of irreversible charge losses can be related to electrolyte decomposition, provided this reaction is the main source of Coulombic inefficiency, which is especially valid for LiFePO_4_-based battery cells. However, nowadays, the protecting character of the SEI formed in the state-of-the-art carbonated-based electrolyte is high, so Coulombic efficiencies cannot be measured with sufficient precision to elucidate differences in the protecting character of different SEI films without a high-precision coulometry system proposed by Dahn et al.^22−24^ Due to the lack of analytical techniques able to provide this type of information, in the last few years, scanning electrochemical microscopy (SECM) in feedback mode has emerged as a powerful tool to evaluate the protecting character of the SEI.^25,26^ To date, this is the only technique that provides in situ information about this very important property of the SEI. Nevertheless, this is a sophisticated technique that operates at the microscale. Additionally, the SECM requires open cells using milliliters of electrolyte (instead of a battery-type cell) and is unavailable in most battery research laboratories. The working principle is based on the addition of a redox mediator in the electrolyte to locally probe the protecting character of the SEI.

**Figure 2:**
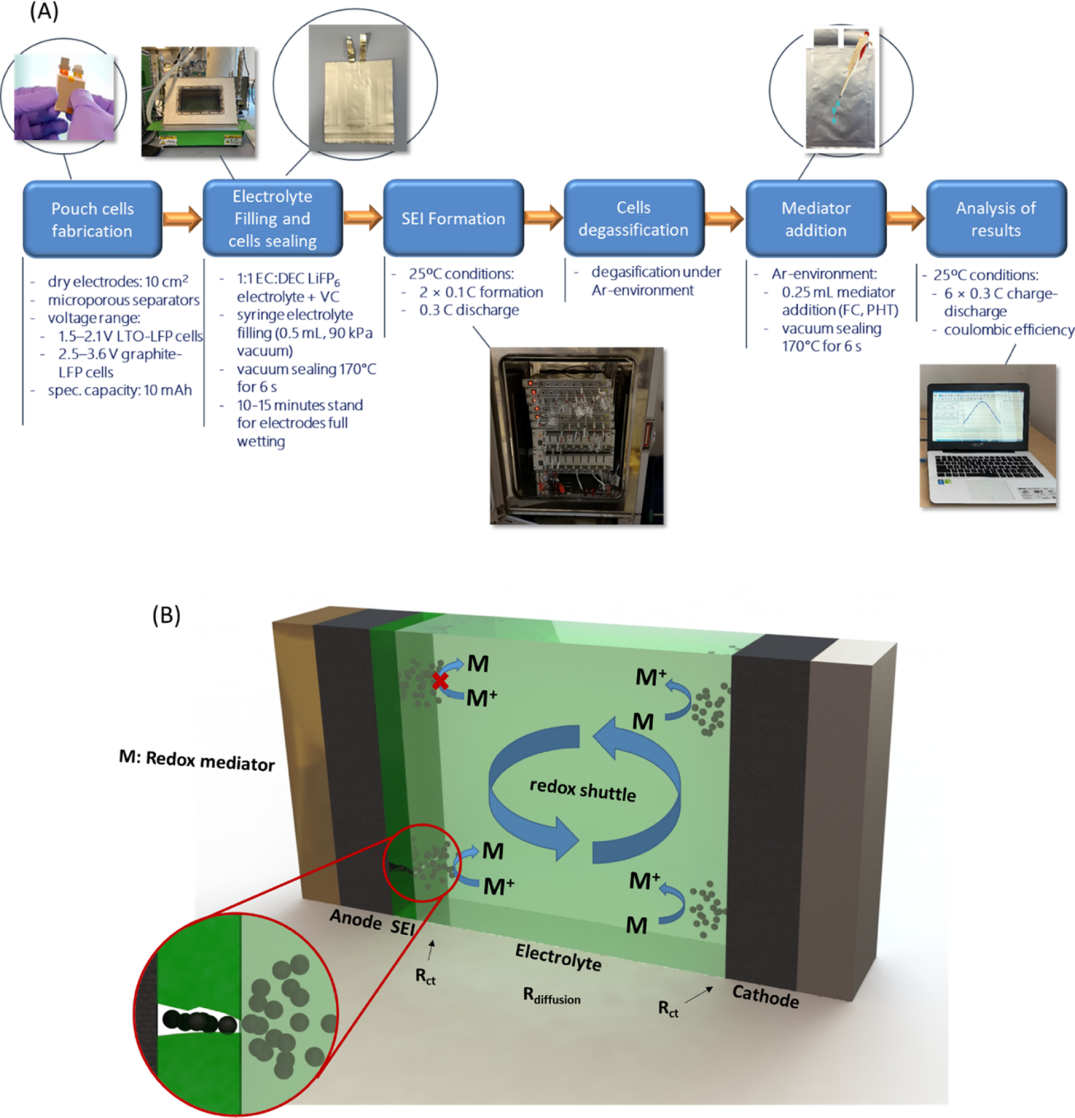
(A) Flow diagram of the steps for the redox-mediated enhanced coulometry measurements. (B) Scheme of a self-discharge process driven by the action of the redox mediator dissolved in the electrolyte.

In this work, we propose the use of redox mediators in full battery cells to probe the overall protecting character of the SEI, which is a key parameter in the resulting cycle life. The testing procedure is shown in Figure 2A. The battery cell is assembled normally and subjected to a standard SEI formation step. Then, during the degassing step, the redox mediator is injected into the cell. After that, the cell is normally cycled to analyze the Coulombic efficiencies during the first five cycles. The working principle by which the presence of the redox mediator enables the assessment of the protecting character of the SEI via an enhanced sensibility of the Coulombic efficiency is as follows (Figure 2B). The redox mediator is oxidized at the positive electrode, diffusing to the negative electrode. If the SEI is not protecting, the redox mediator is reduced at the negative electrode, diffusing back to the positive electrode. The shuttle effect enabled by the lack of the protecting character of the SEI consumes charges, decreasing the Coulombic efficiencies. In other words, if the SEI is not protecting, the battery cell undergoes an internal self-discharge process by the action of the redox mediator, which acts as a “molecular wire” carrying charges between electrodes. In an equivalent circuit, the “resistance” of this molecular wire consists of the addition of several resistances in series, i.e., (i) charge-transfer resistance between the redox mediator and positive electrode, (ii) diffusion resistance (concentration gradient) for the redox mediator between the two electrodes, and (iii) charge-transfer resistance between the redox mediator and negative electrode. While the two former resistances can be assumed to be constant for comparable cells (e.g., graphite–LiFePO_4_ cell using carbonate-based electrolytes), the flow of charges through this internal circuit (self-discharge rate) is determined by the conducting character of the SEI (charge-transfer resistance between redox mediator and electrode through the SEI). As a result, the presence of the redox mediator for battery cells in which the SEI possesses an excellent protecting character does not lead to any changes in the electrochemical performances, i.e., lack of self-discharge process and high Coulombic efficiency. On the contrary, the presence of a poorer protecting SEI leads to an increase in self-discharge and a decrease in Coulombic efficiency.

**Figure 3:**
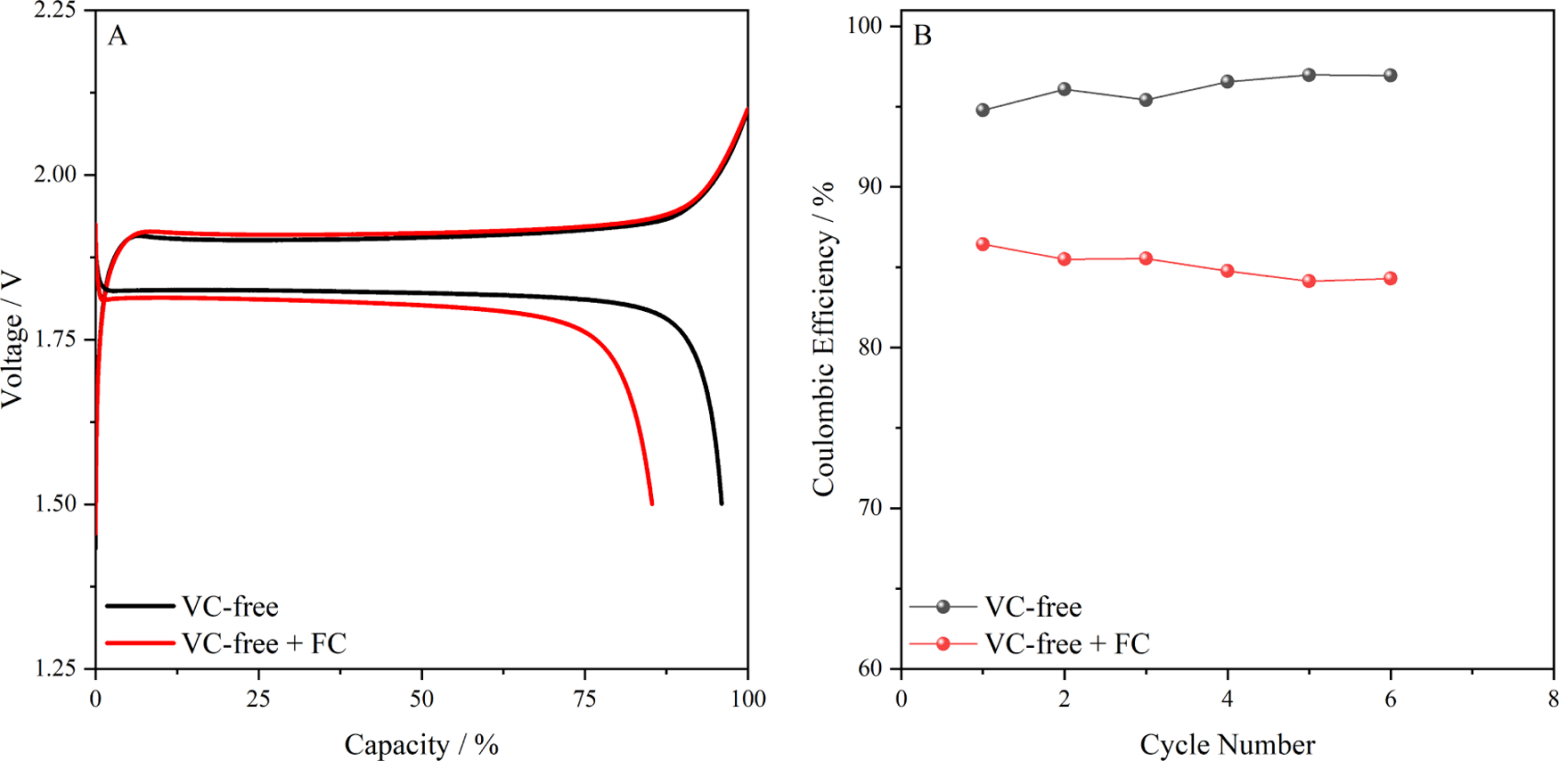
(A) Voltage profiles of LTO–LFP battery cells in the presence and absence of a redox mediator. (B) Evolution of the Coulombic efficiency with the number of cycles in the presence and absence of a redox mediator (0.01 M ferrocene).

To illustrate this latter case, a redox mediator (ferrocene, FC) was added to the state-of-the-art electrolyte without electrolyte additives (1 M LiPF_6_ in EC/DEC) for a Li_4_Ti_5_O_12_–LiFePO_4_ (LTO–LFP) cell. Since both active materials operate within the stability window of the electrolyte,^27^ the formation of an effective and protecting SEI on Li_4_Ti_5_O_12_ does not occur.^26^Figure 3A shows the voltage profile of an LTO–LFP battery cell in the absence and in the presence of a redox mediator galvanostatically cycled at 0.3 C. The profiles are comparable to those reported in the literature for this battery chemistries.^28−31^ The capacity in the first discharge step in the absence of a redox mediator was 8.5 mAh, which is close to the theoretical value of 10 mAh (1 mAh cm^–2^). The only significant difference in the voltage profile related to the presence of a redox mediator is the longer charge process and the short discharge step. As a result, the Coulombic efficiency drastically decreased when a redox mediator was added to the electrolyte (Figure 3B). As hypothesized, the presence of a redox mediator leads to a self-discharge of the battery and a reduction in Coulombic efficiency. These results not only illustrate the working principle of the proposed accelerated redox-enhanced coulometry method but also confirm the lack of the protecting character of any film formed on the surface of LTO electrodes when operated in standard voltage ranges (e.g., 2.1/1.5 V for LTO–LFP).

### 3.2 Graphite–LiFePO_4_ (Gr–LFP) Battery Cells

**Figure 4:**
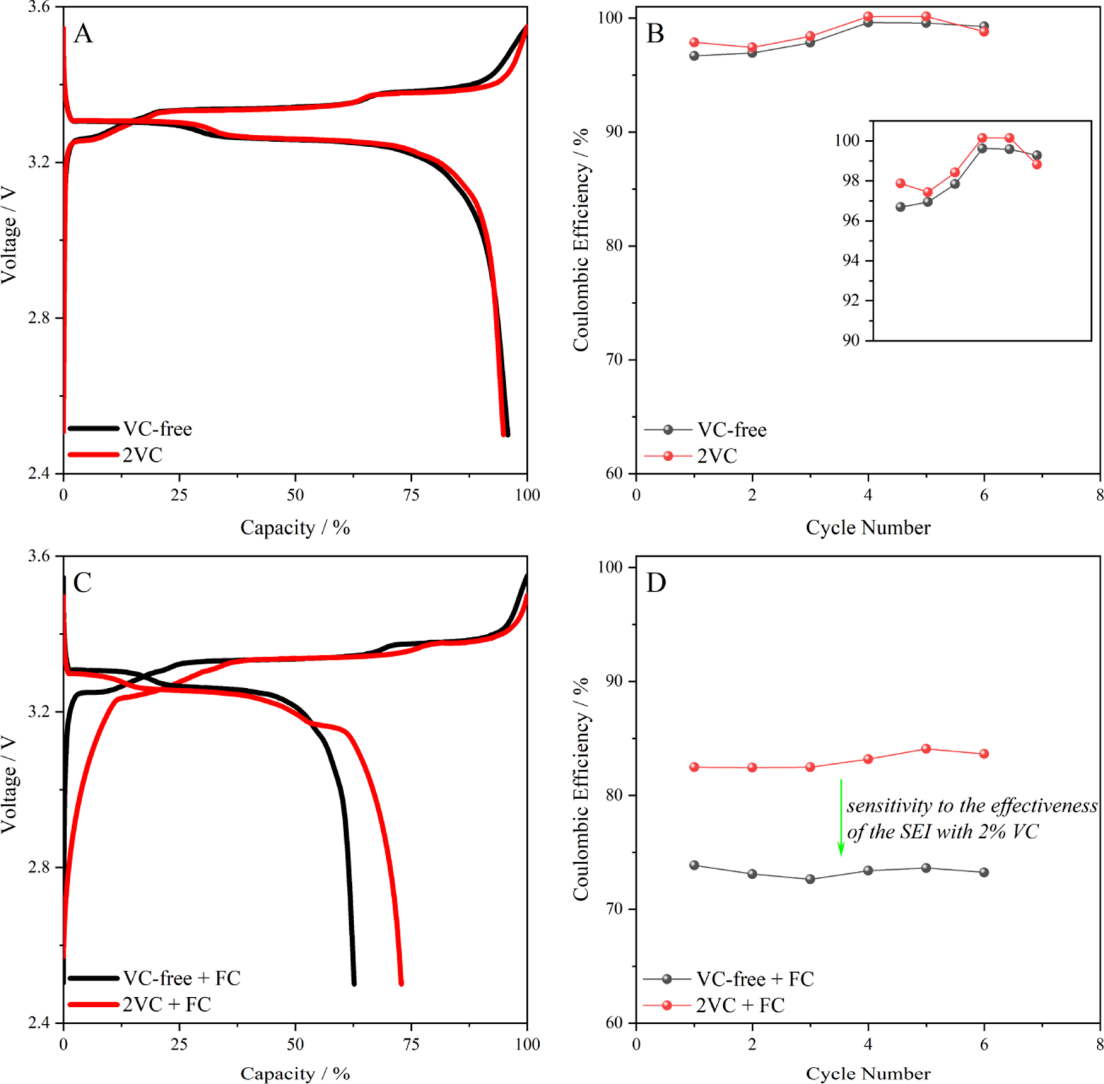
(A) Voltage profiles of Gr–LFP battery cells in the absence of a redox mediator for a VC-free electrolyte and a VC-containing electrolyte (2 wt %). (B) Evolution of the Coulombic efficiency with the number of cycles in the absence of a redox mediator for a VC-free electrolyte and a VC-containing electrolyte (2 wt %). (C) Voltage profiles of Gr–LFP battery cells in the presence of a redox mediator (0.01 M ferrocene) for a VC-free electrolyte and a VC-containing electrolyte (2 wt %). (D) Evolution of the Coulombic efficiency with the number of cycles in the presence of a redox mediator (0.01 M ferrocene) for a VC-free electrolyte and a VC-containing electrolyte (2 wt %).

After the validation of the self-discharge process driven by the redox mediator for a negative electrode that operates at relatively high redox potentials (1.55 V vs Li/Li^+^), a methodology was used to evaluate the protecting character of the SEI formed on graphite electrodes that operate at very cathodic potentials (0.1 V vs Li/Li^+^). Under these reducing conditions, the protecting character of the SEI became of key importance to preventing continuous decomposition of the electrolyte. It should be noted that all cells were subjected to an SEI formation protocol to improve the quality of the SEI, regardless of whether a redox mediator was subsequently added or not. Figure 4A shows the voltage profile of Gr–LFP battery cells in absence of a redox mediator. As for the previous case, the voltage profiles were comparable to those reported in the literature. Note that the shape was dominated by the potential profile of graphite, revealing three plateaus related to the stage of lithiation of graphite.^32^ The influence of vinylene carbonate (VC) in the electrolyte was chosen as a case study since VC is known to act as an SEI-forming additive.^33^ Several studies reported the enormous benefits of the addition of VC in a standard electrolyte in the cycle life of the battery cells.^34,35^ Despite the huge improvement in the long-term cycling stability due to the presence of VC in the electrolyte, the differences in the Coulombic efficiency of VC-free and VC-containing electrolytes were small (Figure 4B). As a result, high-precision coulometry systems have been used to study advanced electrolyte additives and predict improvements in cycle life from the results of a few cycles.^36^ Ideally, an accelerated method should significantly enhance the differences between VC-free and VC-containing electrolytes. Thus, we explore the ability of the redox-mediated enhanced coulometry method to differentiate the performance of cells having VC-free and VC-containing electrolytes. Figure 4C shows the voltage profiles for Gr–LFP cells having VC-free and VC-containing electrolytes in the presence of a redox mediator (ferrocene). The voltage profiles were very similar to those reported in the absence of a redox mediator. The presence of a redox mediator led to a decrease in the Coulombic efficiencies for both cells (Figure 4D). More importantly, the differences related to the presence of VC as an electrolyte additive were significantly enhanced. By the action of the redox mediator, the protecting character of the SEI formed for VC-containing electrolyte was clearly and unambiguously determined since the presence of VC led to an increase of 10% (easily measurable) in the Coulombic efficiency in comparison to the cell having a VC-free electrolyte.

**Figure 5:**
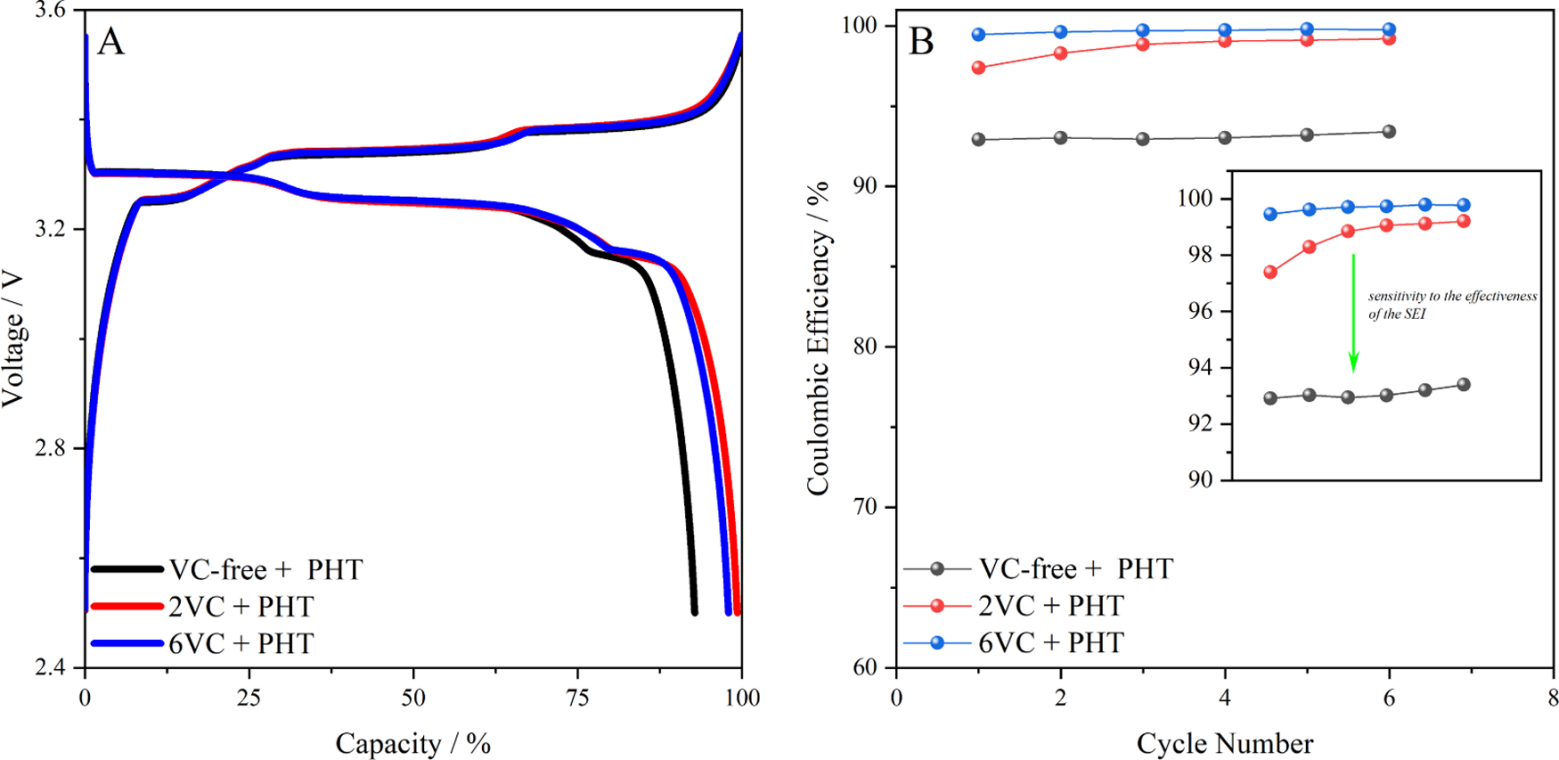
(A) Voltage profiles of Gr–LFP battery cells in the presence of PHT as a redox mediator. (B) Evolution of the Coulombic efficiency with the number of cycles in the presence of 0.01 M PHT as a redox mediator for a VC-free electrolyte, 2 wt % VC-containing electrolyte, and 6 wt % VC-containing electrolyte.

The redox potential of the redox mediator plays a role in the enhancement of Coulombic inefficiency. Since the redox potential of ferrocene is ca. 3.3 V vs Li/Li^+^ (Figure S1) and the delithiation of graphite occurs at 0.1–0.2 V vs Li/Li^+^,^32,37^ the driving force for the charge transfer through the SEI is almost 3 V. This large potential difference enhances the kinetics of charge transfer across the SEI, allowing easy determination of the protecting character of the SEI. Since electrolyte decomposition takes place at 1.2–0.8 V vs Li/Li^+^,^38,39^^38,39^ one can also question whether an excessive driving force induces artefacts. Therefore, we investigate the use of redox mediators having more cathodic potentials. The redox potential of *n*-methyl phthalimide (PHT) is ca. 1.5 V vs Li/Li^+^ (Figure S1), which is a suitable value since the reduction of PHT should thermodynamically take place at a lithiated graphite electrode while its redox potential is closer to the potential of electrolyte decomposition. In addition, the influence of a higher concentration of VC (6 wt %) in the electrolyte was evaluated. Figure 5A shows that the presence of PHT as a redox mediator does not induce relevant changes in the voltage profile of Gr–LFP battery cells. The evolution of Coulombic efficiency as a function of the concentration of VC (additive electrolyte) confirmed that PHT is also able to enhance the difference originating from the protecting character of the SEI. The results indicate that an increase from 2 to 6 wt % still leads to an improved protecting character of the resulting SEI. The presence of a redox mediator can provide additional information, e.g., for how many cycles the SEI formation continues. Figure S2 shows the evolution of the Coulombic efficiencies in the presence of PHT for 20 cycles. In the absence of VC, the SEI formation continues for more than 15 cycles, as the Coulombic efficiency steadily increased with cycling. As the concentration of VC increases, the Coulombic efficiency reached a stable value faster. These results also reveal that the cells are stable for at least few days in the presence of a mediator.

**Figure 6:**
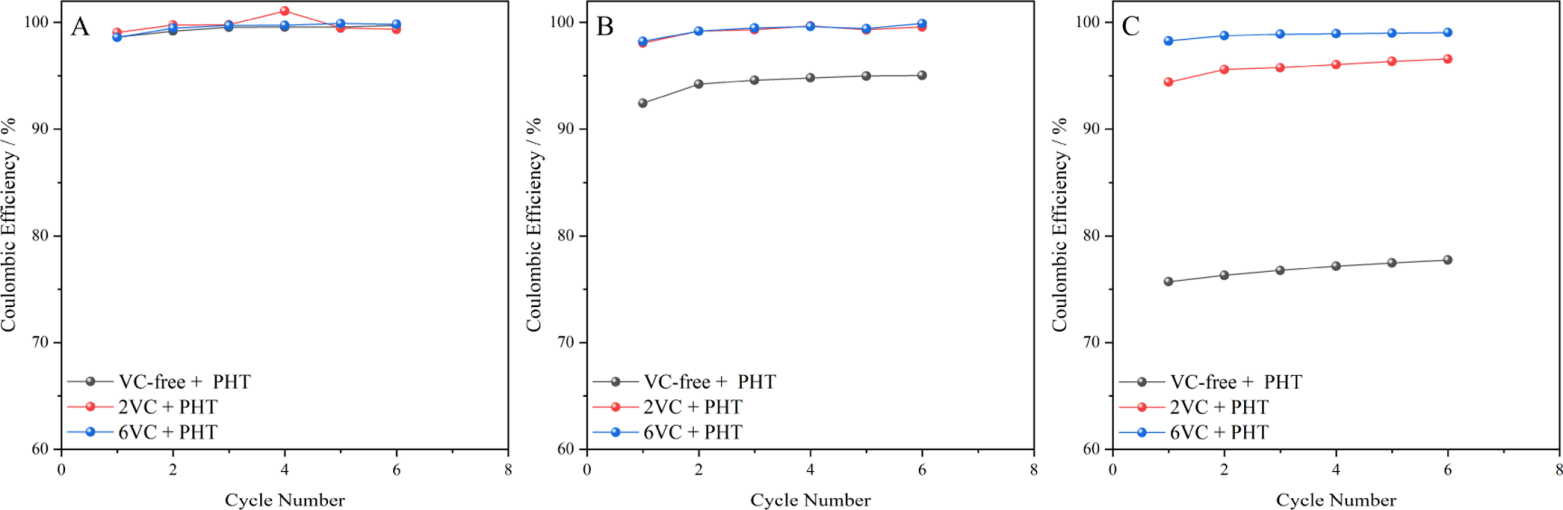
Evolution of the Coulombic efficiencies with the number of cycles for Gr–LFP cells cycled at 1 C varying with the content of VC in the electrolyte: 0, 2, and 6 wt %, (A) in the absence of a redox mediator (PHT), (B) in the presence of 0.01 M PHT, and (C) in the presence of 0.1 M PHT in the electrolyte.

Optimization of cycling conditions can lead to improved differentiation between samples. The introduction of a resting time (open-circuit potential) during which the cell is able to self-discharge can be useful to enhance differences (Figure S3). However, leaving cells at open-circuit voltage is time-consuming, which should be avoided for an accelerated test. Thus, we explored other two parameters to further accelerate the test; higher C-rates and higher concentrations of the redox mediator. At a fixed C-rate of 1 C (Figure 6), the influence of the concentration of redox mediator was evaluated for three electrolyte compositions (VC: 0, 2, and 6 wt %). Note that the C-rate during the SEI formation process was not changed with respect to previous experiments. In the absence of PHT (Figure 6A), the differences in Coulombic efficiencies were not measurable. Using 0.01 M PHT at 1 C (Figure 6B), the difference between VC-free and VC-containing electrolytes was still significant, while Coulombic efficiencies for cells with different VC contents (2 and 6 wt %) were too similar, which is attributed to the faster charge/discharge process compared to 0.3 C. In that case, an increase in the concentration of redox mediator from 0.01 to 0.1 M led to easy differentiation between the three samples (Figure 6C). Although the cells had less time for the self-discharge process (2 h at 1 C versus 6 h at 0.3 C for a full cycle), the increase in the concentration of PHT enhances the self-discharge process, enabling a clear and unambiguous differentiation at 1 C. Thus, it can be concluded that further optimization of the testing conditions can lead to (i) a faster-accelerated assessment and (ii) an unambiguous classification of SEI films having small differences in protecting properties. Also, it should be noted that the increasing protecting character observed for the increasing content of VC is consistent with previous studies. As a matter of fact, Dahn et al. reported a continuous increase in the cycle stability with the increasing content of VC in the range of 0–6 wt %.^40^ While other parameters in addition to the protecting character of the SEI can influence the cycle stability, the correlation of our findings with those reported in the literature is rather encouraging.

## 4 Conclusions

High-precision coulometry system is a powerful tool to predict the cycle stability of high-performing Li-ion batteries within a few hours instead of months. However, this technique is not available in most battery research laboratories. Following this idea of relating the Coulombic efficiency to the effectiveness of the SEI, a low-cost and easily implementable methodology based on enhanced coulometry was proposed in our work. The addition of a redox mediator (soluble redox species) in the electrolyte during the degassing step (after the SEI formation process) leads to a self-discharge process, which is dependent on the protecting character of the SEI: poorly protecting SEI films allow charge transfer across the SEI, increasing the self-discharge rate, while highly protecting SEI prevent self-discharge. As a result, the Coulombic efficiency of a full cycle is decreased by the action of the redox mediator, and the value of the Coulombic efficiency is related to the protecting character of the SEI. Using the well-known positive effect of the VC (SEI-forming additive) as a case study, it was demonstrated that the technique enables unambiguous differentiation of properties of the SEI, which is not possible in the absence of the redox mediator. In addition, the sensitivity of the method can be tuned by adjusting several parameters, e.g., the redox mediator (its redox potential), its concentration, and the C-rate at which the cell is cycled. This methodology not only opens up the possibility of accelerating significantly the search for an electrolyte additive for high-performing Li-ion batteries, which would take months to be tested, but it can provide information about the fundamental understanding of the SEI, e.g., when and how the SEI losses effectiveness. A future combination of the proposed methodology with other sensitive electronics, such as leakage current measurement device or high-precision coulometry system, will surely boost the sensitivity of the assessment at the expense of accessibility for most of the battery research laboratories.

## Supporting Information Available

Experimental details, including cyclic voltammetries of redox mediators versus Li/Li^+^ and lithiation–delithiation processes of graphite, and additional experiments such as SEI evolution and OCV experiment (PDF)
