# Supplementary material for: New Technique for Probing the Protecting Character of the Solid Electrolyte Interphase as a Critical but Elusive Property for Pursuing Long Cycle Life Lithium-Ion Batteries
Source: ACS Appl Mater Interfaces. 2022 Sep 16;14(38):43319–27. doi: 10.1021/acsami.2c11992 (PMC9523620; doi:10.1021/acsami.2c11992)
Supplement: Supplementary file 1 — am2c11992_si_001.pdf [file am2c11992_si_001.pdf]

Supporting Information

A New Technique for Probing the Effectiveness of the Protecting Character of the Solid Electrolyte Interphase as Critical but Elusive Property for Pursuing Long Cycle-Life Lithium-ion Batteries

*Enrique Garcia-Quismondo,1 Sandra Alvarez-Conde,1 Guzmán Garcia,1 Jesús I. Medina-Santos,1 Jesús Palma,1 and Edgar Ventosa 2,3,**

1. Electrochemical Processes Unit, IMDEA Energy, Avda. Ramón de la Sagra 3, 28935, Móstoles, Madrid, Spain

2. Department of Chemistry University of Burgos, Plaza Misael Bañuelos s/n E-09200 Burgos, Spain

3. International Research Centre in Critical Raw Materials-ICCRAM, University of Burgos, Plaza Misael Bañuelos s/n, E-09001, Burgos, Spain.

E-mail: eventosa@ubu.es

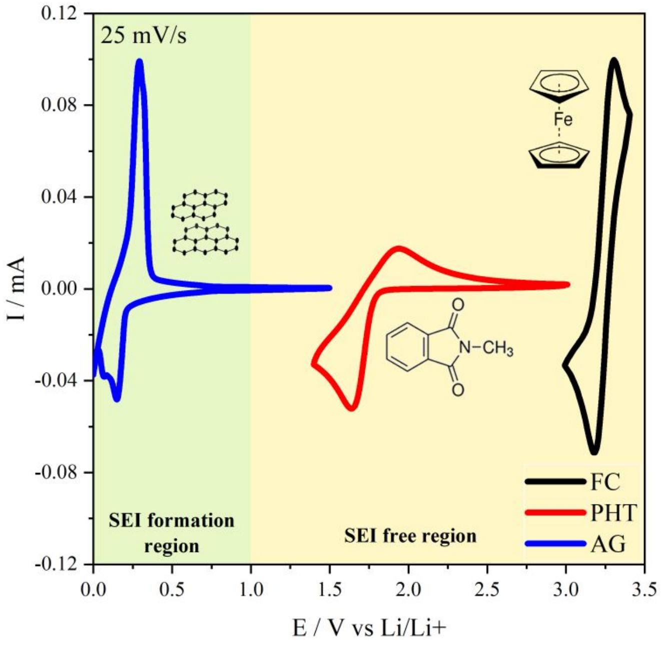

Figure S1. Cyclic Voltammetries of redox-mediators versus vs Li/Li^{+} and lithiation - delithiation processes of graphite consistent with previous reports [1, 2].

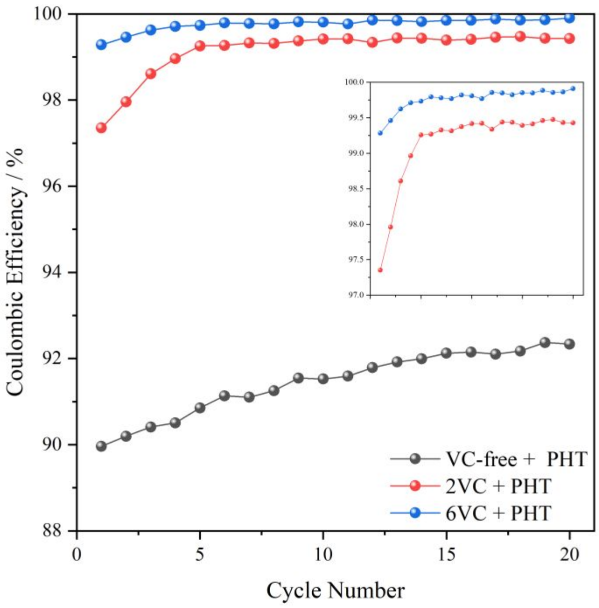

Figure S2. Coulombic efficiencies of Gr-LFP battery cells in the presence of 0.01 M PHT as redox mediator for 20 cycles, for an VC-free electrolyte, 2 wt.% VC-containing electrolyte and 6 wt.% VC-containing electrolyte.

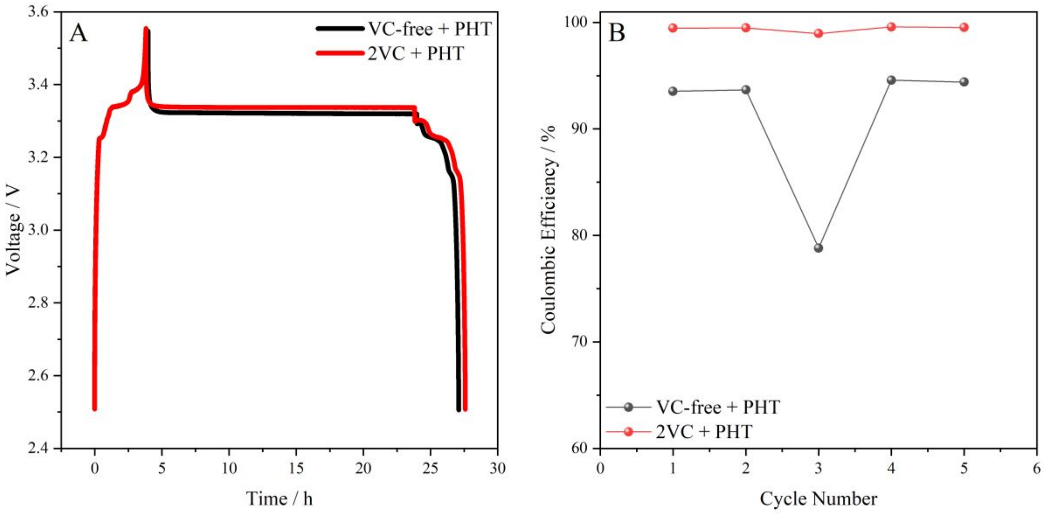

Figure S3. (A) Evolution of cell voltage for Gr-LFP battery cells during a resting time

(open circuit potential) and (B) its resulting coulombic efficiency in the presence of 0.01 M PHT, for an VC-free electrolyte and 2 wt.% VC-containing electrolyte

Supplementary Information References

[1] S. Zhang, M. S. Ding, K. Xu, J. Allen, and T. R. Jow, “Understanding solid electrolyte interface film formation on graphite electrodes,” Electrochem. Solid-State Lett., vol. 4, no. 12, pp. 206–208, 2001.

[2] A. Manthiram and C. Tsang, “Electrode materials for rechargeable lithium batteries,” An. des la Asoc. Quim. Argentina, vol. 84, no. 3, pp. 265–270, 1996.
